# Reply to: Religion and health: not always good

**DOI:** 10.31744/einstein_journal/2020CE6170

**Published:** 2020-11-25

**Authors:** Marco Akerman, Rosilda Mendes, Samira Lima da Costa, Henrique Leonardo Guerra, Rafael Afonso da Silva, Daniele Pompei Sacardo, Juan Carlos Aneiros Fernandez

**Affiliations:** 1 Universidade de São Paulo Faculdade de Saúde Pública São PauloSP Brazil Faculdade de Saúde Pública, Universidade de São Paulo, São Paulo, SP, Brazil.; 2 Universidade Federal de São Paulo SantosSP Brazil Universidade Federal de São Paulo, Santos, SP, Brazil.; 3 Universidade Federal do Rio de Janeiro Rio de JaneiroRJ Brazil Universidade Federal do Rio de Janeiro, Rio de Janeiro, RJ, Brazil.; 4 Pontifícia Universidade Católica de Minas Gerais Belo HorizonteMG Brazil Pontifícia Universidade Católica de Minas Gerais, Belo Horizonte, MG, Brazil.; 5 Universidade Estadual de Campinas Faculdade de Ciências Médicas CampinasSP Brazil Faculdade de Ciências Médicas, Universidade Estadual de Campinas, Campinas, SP, Brazil.

Dear Editor,

The author of the letter aims to make explicit, through the evidence represented by the volume of publications, that religious beliefs can be “bad for your health.” We cannot refute such a statement. However, accepting it does not imply refusing the possibility that religion and religiosity act as protective factors. Religion, like other complex social practices, generally does not respect the dichotomies established by the reductions in our masterful ways of thinking.

As Latour^(^^1^^)^ points out, “a modern is someone who believes that others believe. (…) The modern believe in the belief to understand others (…)”. In dealing with “religious beliefs,” the letter's author reveals his own “beliefs” in the systematic review evidence, while ignoring how this volume of evidence was produced. If we can invite an Islamic feminist, Sibai,^(^^2^^,^^3^^)^ to this conversation (perhaps the author of the letter considers that, just as religion cannot function as a health protective factor, an Islamic woman cannot produce assertions that should be taken seriously), it is worth remembering that, when analyzing the results expressed by the academic production, it is necessary to consider the intersection between “who can speak,” “what one can speak about,” and “in what terms one can speak”.^(^^2^^)^ In the preference given to certain topics (blood transfusion, vaccination, etc.), and to certain religious expressions (fundamentalists), as well as in the establishment of a (supposedly neutral) place from which to judge religious practices (Western scientific medicine), other religious agencies and their effects within the religious-health interface are made invisible. The vitiation of variables and evaluation parameters can then produce, in the balance of published “evidence”, the colonial object (in the sense of being a product of epistemological coloniality) religion-as-something-bad-for-your-health.

We can admit that religious fundamentalism probably produces more harmful than salutary effects, in its freezing of identities and the possibilities of production of agencies in the world. Nevertheless, the equation religion = fundamentalism is “against all evidence and all facts.” It stems from a tendency to accuse of fundamentalism all social practices not subordinate to the prescriptions of Western hegemonic definitions. This tendency is itself fundamentalist, a product of the epistemological fascism exercised based on science, or perhaps it is better to say, of a “bad science,” incapable of recognizing that its own place of enunciation is situated, and thus, of assuming the need to relocate its presuppositions in an epistemologically plural context.

From our article, one cannot produce a generalizing affirmation, such as religion and religiosity are, regardless of the context, a protective factor of health. However, based on specific research and leaving the field of vicious variables we mentioned, it was possible to identify contexts in which religion and religiosity act as health protection factors, contexts in which religion does not act as a way to freeze identities and possibilities of agency production in the world, but as a space for creative construction of identities and multiplication of agencies, favoring the production of healthy subjectivities. We know, however, that in order to accept this alternative, the readers must abandon, themselves, their desire to freeze the identities and possibilities of others, in an essentialist and homogenizing discourse, and recognize the diversity and social complexity of the religious phenomenon.
