# Supplementary material for: Reply to: Religion and health: not always good
Source: Einstein (Sao Paulo). 2020 Nov 25;18:eCE6170. doi: 10.31744/einstein_journal/2020CE6170 (PMC7690925; doi:10.31744/einstein_journal/2020CE6170)
Supplement: Translation

# Translation: Resposta para: Religião e saúde: nem sempre é bom

**Authors:** Marco Akerman, Rosilda Mendes, Samira Lima da Costa, Henrique Leonardo Guerra, Rafael Afonso da Silva, Daniele Pompei Sacardo, Juan Carlos Aneiros Fernandez

**Affiliations:** 1 São PauloSP Brasil Faculdade de Saúde Pública, Universidade de São Paulo, São Paulo, SP, Brasil.; 2 SantosSP Brasil Universidade Federal de São Paulo, Santos, SP, Brasil.; 3 Rio de JaneiroRJ Brasil Universidade Federal do Rio de Janeiro, Rio de Janeiro, RJ, Brasil.; 4 Belo HorizonteMG Brasil Pontifícia Universidade Católica de Minas Gerais, Belo Horizonte, MG, Brasil.; 5 CampinasSP Brasil Faculdade de Ciências Médicas, Universidade Estadual de Campinas, Campinas, SP, Brasil.

Caro Editor,

O autor da carta procura explicitar, por meio da evidência representada pelo volume de publicações, que crenças religiosas podem ser “ *bad for your health* ”. Não podemos refutar tal afirmação. Não obstante, aceitá-la não implica recusar a possibilidade de que religião e religiosidade atuem como fatores protetores. A religião, como outras práticas sociais complexas, não respeita, em geral, as dicotomias estabelecidas pelas reduções de nossos modos doutos de pensar.

Como assinala Latour^(^^1^^)^“é moderno aquele que acredita que os outros acreditam. (…) Os modernos acreditam na crença para compreender os outros (…)”. Ao tratar de “crenças religiosas”, o autor da carta revela suas próprias “crenças” nas evidências da revisão sistemática, ao passo que ignora como esse volume de evidência foi produzido. Se pudermos convidar uma feminista islâmica, Sibai,^(^^2^^,^^3^^)^para essa conversa (talvez o autor da carta considere que, assim como religião não pode funcionar como fator protetor da saúde, uma mulher islâmica não pode produzir asserções que se devam levar a sério), vale lembrar que, ao analisar os resultados expressos pela produção acadêmica, é preciso considerar a intersecção entre “quem pode falar”, “sobre o que se pode falar” e “em que termos se pode falar”.^(^^2^^)^Na preferência dada a certos temas (transfusão de sangue, vacinação etc.) e a certas expressões religiosas (fundamentalistas), bem como na fixação de um lugar (supostamente neutro) a partir do qual se julguem as práticas religiosas (a medicina científica ocidental), são invisibilizadas outras agências religiosas e seus efeitos no âmbito da interface religião-saúde. A viciação das variáveis e dos parâmetros de avaliação pode, então, produzir, no balanço das “evidências” publicadas, o objeto colonial (no sentido de ser produto da colonialidade epistemológica) religião-como-algo-ruim-para-sua-saúde.

Podemos admitir que fundamentalismos religiosos, provavelmente, produzam mais efeitos nocivos que salutares, em seu congelamento de identidades e das possibilidades de produção de agências no mundo. Porém a equação religião = fundamentalismo é “ *against all evidence and all facts* ”. Ela decorre de uma tendência a acusar de fundamentalismo todas as práticas sociais não subordinadas às prescrições das definições hegemônicas ocidentais. Essa tendência é ela mesma fundamentalista, um produto do fascismo epistemológico exercido a partir da ciência, ou, talvez, seja melhor dizer, de uma “má ciência”, incapaz de reconhecer que seu próprio lugar de enunciação é situado e, assim, de assumir a necessidade de relocalizar seus pressuspostos em um contexto epistemologicamente plural.

A partir de nosso artigo, não se pode produzir uma afirmação generalizante como: religião e religiosidade são, independentemente do contexto, um fator protetor da saúde. No entanto, a partir de pesquisa específica e saindo do campo das variáveis viciadas que mencionamos, foi possível identificar contextos em que religião e religiosidade atuam como fatores protetores da saúde, contextos em que a religião não atua como um modo de congelar identidades e possibilidades de produção de agências no mundo, mas como um espaço de construção criativa de identidades e de multiplicação de agências, favorecendo a produção de subjetividades saudáveis. Sabemos, porém, que, para aceitar essa alternativa, o leitor deve abandonar, ele mesmo, seu desejo de congelar as identidades e possibilidades de outrem, em um discurso essencialista e homogeneizador, e reconhecer a diversidade e a complexidade social do fenômeno religioso.
